# Supplementary material for: Efficient inference and identifiability analysis for differential equation models with random parameters
Source: PLoS Comput Biol. 2022 Nov 28;18(11):e1010734. doi: 10.1371/journal.pcbi.1010734 (PMC9731444; doi:10.1371/journal.pcbi.1010734)
Supplement: S1 File — (PDF) [file pcbi.1010734.s001.pdf]

Supplementary material for “Efficient inference and identifiability analysis for differential equation models with random parameters”

Alexander P. Browning[1,2,3], Christopher Drovandi[1,2], Ian W. Turner[1], Adrianne L. Jenner[1,2], and Matthew J. Simpson*[1,2]

[1] *School of Mathematical Sciences, Queensland University of Technology, Brisbane, Australia*
[2] *QUT Centre for Data Science, Queensland University of Technology, Brisbane, Australia*
[3] *Mathematical Institute, University of Oxford, Oxford, United Kingdom*

November 17, 2022

Contents

S1 Derivation of moment equations **2**
S1.1 Definitions . . . 2
S1.2 Intermediate results . . . 3
S1.3 Obtaining approximate expressions for the moments of $f(\boldsymbol{\theta})$ . . . 5
S1.3.1 First-order . . . 6
S1.3.2 Second-order . . . 6
S1.3.3 Third-order . . . 8
S1.3.4 Reshaped expressions . . . 9

S2 Kolmogorov-Smirnov test results for Fig 2 **11**

S3 Approximate solutions to random parameter logistic model **12**

S4 Approximate solutions for linear two-pool model **13**

S5 Approximate solutions for non-linear two-pool model **14**

S6 Additional comparison for misspecified bimodal model **15**

S7 Bistable model **16**

S8 Uniformly distributed parameters model **17**

*Corresponding author: matthew.simpson@qut.edu.au

S1 Derivation of moment equations

S1.1 Definitions

Definition 1. (Frobenius inner product) *The Frobenius inner product, $\circ$, is a binary operator that yields the sum of the component-wise product of two tensors A and B of the same size and shape given by*

$$A \circ B = \sum_{i_1} \sum_{i_2} \cdots \sum_{i_k} A_{i_1,i_2,\ldots,i_k} B_{i_1,i_2,\ldots,i_k} = \mathrm{vec}(A)^\intercal \mathrm{vec}(B).$$

Here, $\mathrm{vec}(A)$ denotes the vectorisation operator, returning a vector containing all the elements of A in column-major order.

If A and B are vectors, then the Frobenius inner product reduces to the dot product, such that $A \circ B = A^\intercal B = A \cdot B$. If A and B are matrices, then $A \circ B = \mathrm{Tr}(A^\intercal B)$.

Definition 2. (Observed moments) *The k-th order observed moment of the vector $\boldsymbol{\varphi} \in \mathbb{R}^d$ is*

$$M_k(\boldsymbol{\varphi}) \in \mathbb{R}^{d^k}.$$

and contains elements relating to all possible k-term products of the elements of $\boldsymbol{\varphi}$. For example, we might define M_k recursively where

$$\begin{aligned}
M_0(\boldsymbol{\varphi}) &= 1, \\
M_1(\boldsymbol{\varphi}) &= \boldsymbol{\varphi}, \\
M_2(\boldsymbol{\varphi}) &= \boldsymbol{\varphi} \otimes \boldsymbol{\varphi} = \mathrm{vec}(\boldsymbol{\varphi}\boldsymbol{\varphi}^\intercal), \\
M_k(\boldsymbol{\varphi}) &= \boldsymbol{\varphi} \otimes M_{k-1}(\boldsymbol{\varphi}) = M_{k-1}(\boldsymbol{\varphi}) \otimes \boldsymbol{\varphi} && k \geq 1, \\
M_k(\boldsymbol{\varphi}) &= M_{k-a}(\boldsymbol{\varphi}) \otimes M_a(\boldsymbol{\varphi}), && k \geq a.
\end{aligned}$$

Regardless of the shape of $M_k(\boldsymbol{\varphi})$, for $\boldsymbol{\varphi} = \boldsymbol{\theta} - \hat{\boldsymbol{\theta}}$, the expectations of $M_k(\boldsymbol{\theta} - \hat{\boldsymbol{\theta}})$ relate to the covariance matrix, coskewness tensor and cokurtosis tensor for $k = 2$, 3 and 4, respectively for $\hat{\boldsymbol{\theta}} = \mathbb{E}(\boldsymbol{\theta})$. We denote these tensors

$$\begin{aligned}
\mathbb{V}(\boldsymbol{\theta}) &= \langle M_2(\boldsymbol{\theta} - \hat{\boldsymbol{\theta}}) \rangle, \\
\mathbb{S}(\boldsymbol{\theta}) &= \langle M_3(\boldsymbol{\theta} - \hat{\boldsymbol{\theta}}) \rangle, \\
\mathbb{K}(\boldsymbol{\theta}) &= \langle M_4(\boldsymbol{\theta} - \hat{\boldsymbol{\theta}}) \rangle,
\end{aligned}$$

respectively.

Definition 3. (Differential operator) *The k-th order differential operator of the function $f : \mathbb{R}^d \to \mathbb{R}$ is*

$$D^k f = \frac{\partial^k f}{\partial M_k(\boldsymbol{\varphi})}.$$

We can also define the differential operator recursively

$$\begin{aligned}
Df(\boldsymbol{\varphi}) &= \nabla f(\boldsymbol{\varphi}), \\
D^2 f(\boldsymbol{\varphi}) &= \nabla \otimes \nabla f(\boldsymbol{\varphi}), \\
D^k f(\boldsymbol{\varphi}) &= \nabla \otimes D^{k-1} f(\boldsymbol{\varphi}) \in \mathbb{R}^{d^k}, \quad k \geq 2.
\end{aligned}$$

Note that the second-order differential operator is also known as the Hessian operator

$$D^2 f(\boldsymbol{\varphi}) = Hf(\boldsymbol{\varphi}).$$

S1.2 Intermediate results

Proposition 1. *For constant* $A \in \mathbb{R}^{d_1 \times d_2 \times \cdots \times d_n}$ *and function* $B(\boldsymbol{\theta}) : \mathbb{R}^d \to \mathbb{R}^{d_1 \times d_2 \times \cdots \times d_n}$*, where* $\boldsymbol{\theta}$ *is a random vector,*

$$\langle A \circ B(\boldsymbol{\theta}) \rangle = A \circ \langle B(\boldsymbol{\theta}) \rangle,$$

where $\langle \cdot \rangle$ denotes an expectation with respect to $\boldsymbol{\theta}$.

Proof.

$$\begin{aligned}
\langle A \circ B(\boldsymbol{\theta}) \rangle &= \langle \text{vec}(A)^\intercal \text{vec}(B(\boldsymbol{\theta})) \rangle, \\
&= \text{vec}(A)^\intercal \langle \text{vec}(B(\boldsymbol{\theta})) \rangle, && \text{since } \mathbb{E}_X(AX) = A\mathbb{E}_X(X) \text{ by [1,2]}, \\
&= \text{vec}(A) \circ \langle \text{vec}(B(\boldsymbol{\theta})) \rangle, \\
&= A \circ \langle B(\boldsymbol{\theta}) \rangle.
\end{aligned}$$

□

Proposition 2. *For symmetric matrix* $A \in \mathbb{R}^{d \times d}$ *and vectors* $\mathbf{x}, \mathbf{y} \in \mathbb{R}^d$*,*

$$\mathbf{x}^\intercal A \mathbf{y} = A \circ \mathbf{y}\mathbf{x}^\intercal = A \circ \mathbf{x}\mathbf{y}^\intercal.$$

Proof.

$$\begin{aligned}
A \circ \mathbf{y}\mathbf{x}^\intercal &= \text{Tr}(A^\intercal \mathbf{y}\mathbf{x}^\intercal), \\
&= \text{Tr}((A\mathbf{y})\mathbf{x}^\intercal), \\
&= \text{Tr}(\mathbf{x}^\intercal (A\mathbf{y})), \\
&= \mathbf{x}^\intercal A \mathbf{y}.
\end{aligned}$$

Also, consider $A \circ \mathbf{y}\mathbf{x}^\intercal = A^\intercal \circ (\mathbf{y}\mathbf{x}^\intercal)^\intercal = A \circ \mathbf{x}\mathbf{y}^\intercal$. □

Proposition 3. *For matrices* $A, B \in \mathbb{R}^{m \times n}$ *and* $C, D \in \mathbb{R}^{p \times q}$*,*

$$(A \circ B)(C \circ D) = (A \otimes C) \circ (B \otimes D).$$

Proof.

$$\begin{aligned}(A \circ B)(C \circ D) &= \mathrm{Tr}(A^\intercal B)\,\mathrm{Tr}(C^\intercal D),\\ &= \mathrm{Tr}\left((A^\intercal B) \otimes (C^\intercal D)\right),\\ &= \mathrm{Tr}\left((A^\intercal \otimes C^\intercal)(B \otimes D)\right),\\ &= \mathrm{Tr}\left((A \otimes C)^\intercal (B \otimes D)\right),\\ &= (A \otimes C) \circ (B \otimes D).\end{aligned}$$

□

Proposition 4. (Multivariate Taylor series) *Let $f : \mathbb{R}^d \to \mathcal{R} \subset \mathbb{R}$ and let D^n and M_n be defined as in definitions 2 and 3, respectively. Then*

$$f(\boldsymbol{a}+\boldsymbol{h}) = \sum_{k=0}^{\infty} \frac{1}{k!} D^k f(\boldsymbol{a}) \circ M_k(\boldsymbol{h}).$$

for $\boldsymbol{a}, \boldsymbol{h} \in \mathbb{R}^d$.

Proof. First, consider the scalar function $F(t) = f(\boldsymbol{a} + t\boldsymbol{h})$ such that

$$F(t) = \sum_{k=0}^{\infty} \frac{1}{k!} F^{(k)}(0)\, t^k. \tag{S1}$$

Let $\boldsymbol{r}(t) = \boldsymbol{a} + t\boldsymbol{h}$ and consider

$$F'(t) = \frac{\mathrm{d}f(\boldsymbol{r}(t))}{\mathrm{d}t} = \frac{\partial \boldsymbol{r}(t)}{\partial t} \cdot \nabla f(\boldsymbol{r}(t)) = (\boldsymbol{h} \cdot \nabla) f(\boldsymbol{r}(t)),$$

by the chain rule. Observe further that

$$\begin{aligned}F''(t) &= (\boldsymbol{h} \cdot \nabla)\frac{\mathrm{d}f(\boldsymbol{r}(t))}{\mathrm{d}t},\\ &= (\boldsymbol{h} \cdot \nabla)(\boldsymbol{h} \cdot \nabla) f(\boldsymbol{r}(t)),\\ &= (\boldsymbol{h} \cdot \nabla)^2 f(\boldsymbol{r}(t)).\end{aligned}$$

Assume now that $F^{(k)}(t) = (\boldsymbol{h} \cdot \nabla)^k f(\boldsymbol{r}(t))$ holds for $k = \ell$, then

$$\begin{aligned}F^{(\ell+1)}(t) &= \frac{\mathrm{d}}{\mathrm{d}t} F^{(\ell)}(t),\\ &= (\boldsymbol{h} \cdot \nabla)^\ell \frac{\mathrm{d}f(\boldsymbol{r}(t))}{\mathrm{d}t},\\ &= (\boldsymbol{h} \cdot \nabla)^\ell (\boldsymbol{h} \cdot \nabla) f(\boldsymbol{r}(t)),\\ &= (\boldsymbol{h} \cdot \nabla)^{\ell+1} f(\boldsymbol{r}(t)),\end{aligned}$$

which completes the induction step. Therefore, by the principle of mathematical induction, $F^{(k)}(t) = (\boldsymbol{h} \cdot \nabla)^k f(\boldsymbol{r}(t))$ holds true for all $k \in \mathbb{N}$ by the principle of mathematical induction.

Thus, eq. (S1) can be expressed in the form

$$F(t) = f(\boldsymbol{a} + \boldsymbol{h}t) = \sum_{k=0}^{\infty} \frac{1}{k!} (\boldsymbol{h} \cdot \nabla)^k f(\boldsymbol{a})\, t^k$$

and for $t = 1$, we have that

$$f(\boldsymbol{a} + \boldsymbol{h}) = \sum_{k=0}^{\infty} \frac{1}{k!} (\boldsymbol{h} \cdot \nabla)^k f(\boldsymbol{a}).$$

It remains to be shown that $(\boldsymbol{h} \cdot \nabla)^k f(\boldsymbol{a}) = D^k f(\boldsymbol{a}) \circ M_k(\boldsymbol{h})$. For $k = 1$, we have that

$$Df(\boldsymbol{a}) \circ M_1(\boldsymbol{h}) = \nabla f(\boldsymbol{a}) \circ \boldsymbol{h} = \boldsymbol{h} \cdot \nabla f(\boldsymbol{a}) = (\boldsymbol{h} \cdot \nabla) f(\boldsymbol{a}).$$

Assuming that $(\boldsymbol{h} \cdot \nabla)^k f(\boldsymbol{a}) = D^k f(\boldsymbol{a}) \circ M_k(\boldsymbol{h})$ holds for $k = \ell$, then

$$\begin{aligned}
(\boldsymbol{h} \cdot \nabla)^{\ell+1} f(\boldsymbol{a}) &= (\boldsymbol{h} \cdot \nabla)(\boldsymbol{h} \cdot \nabla)^{\ell} f(\boldsymbol{a}), \\
&= (\boldsymbol{h} \circ \nabla)(\boldsymbol{h} \circ \nabla)^{\ell} f(\boldsymbol{a}), && \text{by def. 1,} \\
&= (\nabla \circ \boldsymbol{h}) \left(D^{\ell} f(\boldsymbol{a}) \circ M_{\ell}(\boldsymbol{h}) \right), \\
&= \left(\nabla \otimes D^{\ell} f(\boldsymbol{a}) \right) \circ \left(\boldsymbol{h} \otimes M_{\ell}(\boldsymbol{h}) \right), && \text{by prop. 3,} \\
&= D^{\ell+1} f(\boldsymbol{a}) \circ M_{\ell+1}(\boldsymbol{h}), && \text{by defs. 2 and 3,}
\end{aligned}$$

which concludes the induction step. Therefore, $(\boldsymbol{h} \cdot \nabla)^n f(\boldsymbol{a}) = D^n f(\boldsymbol{a}) \circ M_n(\boldsymbol{h})$ holds for all $n \in \mathbb{N}$ by the principle of mathematical induction. □

S1.3 Obtaining approximate expressions for the moments of $f(\boldsymbol{\theta})$

By proposition 4 we have that

$$f(\boldsymbol{\theta}) = f(\hat{\boldsymbol{\theta}}) + \sum_{k=1}^{\infty} \frac{1}{k!} D^k f(\hat{\boldsymbol{\theta}}) \circ M_k(\boldsymbol{\theta} - \hat{\boldsymbol{\theta}}), \tag{S2}$$

where $\boldsymbol{a} = \hat{\boldsymbol{\theta}}$ and $\boldsymbol{h} = \boldsymbol{\theta} - \hat{\boldsymbol{\theta}}$. Truncating the series to $k \leq 2$ yields a second-order approximation to $f(\boldsymbol{\theta})$ about $\boldsymbol{\theta} = \hat{\boldsymbol{\theta}}$ given by

$$f(\boldsymbol{\theta}) \approx f(\hat{\boldsymbol{\theta}}) + \nabla f(\hat{\boldsymbol{\theta}}) \cdot (\boldsymbol{\theta} - \hat{\boldsymbol{\theta}}) + \frac{1}{2} H f(\hat{\boldsymbol{\theta}}) \circ M_2(\boldsymbol{\theta} - \hat{\boldsymbol{\theta}}), \tag{S3}$$

where $\circ$, $M_2(\boldsymbol{\theta})$, and $Hf(\hat{\boldsymbol{\theta}})$ are defined in definitions 1, 2, and 3, respectively.

In the main paper, we present approximate expressions for the mean $\langle f(\boldsymbol{\theta}) \rangle$, the univariate second moments $\langle f^2(\boldsymbol{\theta}) \rangle$, the covariance $\langle f_i(\boldsymbol{\theta}) f_j(\boldsymbol{\theta}) \rangle$ for two functions $f_i(\boldsymbol{\theta})$ and $f_j(\boldsymbol{\theta})$, and finally for the univariate third moments, $\langle f^3(\boldsymbol{\theta}) \rangle$. Here, we derive these expressions using the notation defined in the definitions and results derived in the propositions from section S1.1 and section S1.2. In this supporting information document, we work with the definitions of $M_k(\boldsymbol{\varphi})$ given in definition 2 having the useful property that $M_k(\boldsymbol{\varphi})$ are matrices for all k. In

section S1.3.4, we explain how the results are invariant to the shape of $M_k(\boldsymbol{\varphi})$ and therefore, how the results relate to the formulation in the main text where $M_k(\boldsymbol{\varphi})$ is a k-dimensional tensor. This observation enables the elements of $M_k(\boldsymbol{\varphi})$ (and therefore, elements of the expectation $\langle M_k(\boldsymbol{\varphi})\rangle$) to be more readily obtained.

In the present work, we consider only the quadratic approximation (eq. (S3)) however similar working can be applied for an approximation of any order.

S1.3.1 First-order

Taking expectations of eq. (S3), we have that

$$\begin{aligned}\langle f(\boldsymbol{\theta})\rangle &\approx \langle f(\hat{\boldsymbol{\theta}})\rangle + \left\langle \nabla f(\hat{\boldsymbol{\theta}}) \cdot (\boldsymbol{\theta} - \hat{\boldsymbol{\theta}})\right\rangle + \left\langle \frac{1}{2} H f(\hat{\boldsymbol{\theta}}) \circ M_2(\boldsymbol{\theta} - \hat{\boldsymbol{\theta}})\right\rangle, \\ &= f(\hat{\boldsymbol{\theta}}) + \nabla f(\hat{\boldsymbol{\theta}}) \cdot \left\langle (\boldsymbol{\theta} - \hat{\boldsymbol{\theta}})\right\rangle + \frac{1}{2} H f(\hat{\boldsymbol{\theta}}) \circ \left\langle M_2(\boldsymbol{\theta} - \hat{\boldsymbol{\theta}})\right\rangle, \\ &= f(\hat{\boldsymbol{\theta}}) + \mathbb{V}(\boldsymbol{\theta}) \circ \frac{1}{2} H f(\hat{\boldsymbol{\theta}}).\end{aligned} \tag{S4}$$

S1.3.2 Second-order

It suffices to derive an expression for $\langle f_i(\boldsymbol{\theta}) f_j(\boldsymbol{\theta})\rangle$ since $\langle f^2(\boldsymbol{\theta})\rangle = \langle f(\boldsymbol{\theta}) f(\boldsymbol{\theta})\rangle$. Consider

$$\begin{aligned} f_i(\boldsymbol{\theta}) f_j(\boldsymbol{\theta}) \approx\ & f_i(\hat{\boldsymbol{\theta}}) f_j(\hat{\boldsymbol{\theta}}) + f_i(\hat{\boldsymbol{\theta}}) \nabla f_j(\hat{\boldsymbol{\theta}}) \cdot (\boldsymbol{\theta} - \hat{\boldsymbol{\theta}}) + f_j(\hat{\boldsymbol{\theta}}) \nabla f_i(\hat{\boldsymbol{\theta}}) \cdot (\boldsymbol{\theta} - \hat{\boldsymbol{\theta}}) \\ & + \underbrace{\left[\nabla f_i(\hat{\boldsymbol{\theta}}) \cdot (\boldsymbol{\theta} - \hat{\boldsymbol{\theta}})\right] \left[\nabla f_j(\hat{\boldsymbol{\theta}}) \cdot (\boldsymbol{\theta} - \hat{\boldsymbol{\theta}})\right]}_{\text{Term 1}} \\ & + \frac{1}{2} f_i(\hat{\boldsymbol{\theta}}) H f_j(\hat{\boldsymbol{\theta}}) \circ M_2(\boldsymbol{\theta} - \hat{\boldsymbol{\theta}}) + \frac{1}{2} f_j(\hat{\boldsymbol{\theta}}) H f_i(\hat{\boldsymbol{\theta}}) \circ M_2(\boldsymbol{\theta} - \hat{\boldsymbol{\theta}}) \\ & + \frac{1}{2} \underbrace{\left[\nabla f_i(\hat{\boldsymbol{\theta}}) \cdot (\boldsymbol{\theta} - \hat{\boldsymbol{\theta}})\right] \left[H f_j(\hat{\boldsymbol{\theta}}) \circ M_2(\boldsymbol{\theta} - \hat{\boldsymbol{\theta}})\right]}_{\text{Term 2}} \\ & + \frac{1}{2} \left[\nabla f_j(\hat{\boldsymbol{\theta}}) \cdot (\boldsymbol{\theta} - \hat{\boldsymbol{\theta}})\right] \left[H f_i(\hat{\boldsymbol{\theta}}) \circ M_2(\boldsymbol{\theta} - \hat{\boldsymbol{\theta}})\right] \\ & + \frac{1}{4} \underbrace{\left[H f_i(\hat{\boldsymbol{\theta}}) \circ M_2(\boldsymbol{\theta} - \hat{\boldsymbol{\theta}})\right] \left[H f_j(\hat{\boldsymbol{\theta}}) \circ M_2(\boldsymbol{\theta} - \hat{\boldsymbol{\theta}})\right]}_{\text{Term 3}}. \end{aligned} \tag{S5}$$

The unnamed terms in the expression above relate to terms that already appear in the first-order expression, or terms that appear twice.

We now apply results from propositions 1 to 3 so that expectations related to the moments of $\boldsymbol{\theta}$ can be taken.

Term 1

$$\begin{aligned}\Big[\nabla f_i(\hat{\boldsymbol{\theta}})\cdot(\boldsymbol{\theta}-\hat{\boldsymbol{\theta}})\Big]\Big[\nabla f_j(\hat{\boldsymbol{\theta}})\cdot(\boldsymbol{\theta}-\hat{\boldsymbol{\theta}})\Big] &= \Big[\nabla f_i(\hat{\boldsymbol{\theta}})\circ(\boldsymbol{\theta}-\hat{\boldsymbol{\theta}})\Big]\Big[\nabla f_j(\hat{\boldsymbol{\theta}})\circ(\boldsymbol{\theta}-\hat{\boldsymbol{\theta}})\Big], \\ &= \Big[\nabla f_i(\hat{\boldsymbol{\theta}})\otimes\nabla f_j(\hat{\boldsymbol{\theta}})\Big]\circ\Big[(\boldsymbol{\theta}-\hat{\boldsymbol{\theta}})\otimes(\boldsymbol{\theta}-\hat{\boldsymbol{\theta}})\Big], \\ &= M_2(\boldsymbol{\theta}-\hat{\boldsymbol{\theta}})\circ\Big(\nabla f_i(\hat{\boldsymbol{\theta}})\otimes\nabla f_j(\hat{\boldsymbol{\theta}})\Big). \\ \therefore \Big\langle\Big[\nabla f_i(\hat{\boldsymbol{\theta}})\cdot(\boldsymbol{\theta}-\hat{\boldsymbol{\theta}})\Big]\Big[\nabla f_j(\hat{\boldsymbol{\theta}})\cdot(\boldsymbol{\theta}-\hat{\boldsymbol{\theta}})\Big]\Big\rangle &= \Big\langle M_2(\boldsymbol{\theta}-\hat{\boldsymbol{\theta}})\circ\Big(\nabla f_i(\hat{\boldsymbol{\theta}})\otimes\nabla f_j(\hat{\boldsymbol{\theta}})\Big)\Big\rangle, \\ &= \Big\langle M_2(\boldsymbol{\theta}-\hat{\boldsymbol{\theta}})\Big\rangle\circ\Big(\nabla f_i(\hat{\boldsymbol{\theta}})\otimes\nabla f_j(\hat{\boldsymbol{\theta}})\Big), \\ &= \mathbb{V}(\boldsymbol{\theta})\circ\Big(\nabla f_i(\hat{\boldsymbol{\theta}})\otimes\nabla f_j(\hat{\boldsymbol{\theta}})\Big).\end{aligned}$$

Term 2

$$\begin{aligned}\Big[\nabla f_i(\hat{\boldsymbol{\theta}})\cdot(\boldsymbol{\theta}-\hat{\boldsymbol{\theta}})\Big]\Big[Hf_j(\hat{\boldsymbol{\theta}})\circ M_2(\boldsymbol{\theta}-\hat{\boldsymbol{\theta}})\Big] &= \Big[Hf_j(\hat{\boldsymbol{\theta}})\circ M_2(\boldsymbol{\theta}-\hat{\boldsymbol{\theta}})\Big]\Big[\nabla f_i(\hat{\boldsymbol{\theta}})\cdot(\boldsymbol{\theta}-\hat{\boldsymbol{\theta}})\Big], \\ &= \Big(Hf_j(\hat{\boldsymbol{\theta}})\otimes\nabla f_i(\hat{\boldsymbol{\theta}})\Big)\circ\Big(M_2(\boldsymbol{\theta}-\hat{\boldsymbol{\theta}})\otimes(\boldsymbol{\theta}-\hat{\boldsymbol{\theta}})\Big), \\ &= \Big(Hf_j(\hat{\boldsymbol{\theta}})\otimes\nabla f_i(\hat{\boldsymbol{\theta}})\Big)\circ M_3(\boldsymbol{\theta}-\hat{\boldsymbol{\theta}}). \\ \therefore \Big\langle\Big[\nabla f_i(\hat{\boldsymbol{\theta}})\cdot(\boldsymbol{\theta}-\hat{\boldsymbol{\theta}})\Big]\Big[Hf_j(\hat{\boldsymbol{\theta}})\circ M_2(\boldsymbol{\theta}-\hat{\boldsymbol{\theta}})\Big]\Big\rangle &= \mathbb{S}(\boldsymbol{\theta})\circ\Big(Hf_j(\hat{\boldsymbol{\theta}})\otimes\nabla f_i(\hat{\boldsymbol{\theta}})\Big).\end{aligned}$$

Term 3

$$\begin{aligned}\Big[Hf_i(\hat{\boldsymbol{\theta}})\circ M_2(\boldsymbol{\theta}-\hat{\boldsymbol{\theta}})\Big]\Big[Hf_j(\hat{\boldsymbol{\theta}})\circ M_2(\boldsymbol{\theta}-\hat{\boldsymbol{\theta}})\Big] &= \Big(Hf_i(\hat{\boldsymbol{\theta}})\otimes Hf_j(\hat{\boldsymbol{\theta}})\Big)\circ\Big(M_2(\boldsymbol{\theta}-\hat{\boldsymbol{\theta}})\otimes M_2(\boldsymbol{\theta}-\hat{\boldsymbol{\theta}})\Big), \\ &= \Big(Hf_i(\hat{\boldsymbol{\theta}})\otimes Hf_j(\hat{\boldsymbol{\theta}})\Big)\circ M_4(\boldsymbol{\theta}-\hat{\boldsymbol{\theta}}). \\ \therefore \Big\langle\Big[Hf_i(\hat{\boldsymbol{\theta}})\circ M_2(\boldsymbol{\theta}-\hat{\boldsymbol{\theta}})\Big]\Big[Hf_j(\hat{\boldsymbol{\theta}})\circ M_2(\boldsymbol{\theta}-\hat{\boldsymbol{\theta}})\Big]\Big\rangle &= \mathbb{K}(\boldsymbol{\theta})\circ\Big(Hf_i(\hat{\boldsymbol{\theta}})\otimes Hf_j(\hat{\boldsymbol{\theta}})\Big).\end{aligned}$$

Therefore, we have that

$$\begin{aligned}\langle f_i(\boldsymbol{\theta})f_j(\boldsymbol{\theta})\rangle \approx \Big\langle f_i(\hat{\boldsymbol{\theta}})f_j(\hat{\boldsymbol{\theta}})\Big\rangle &+ \mathbb{V}(\boldsymbol{\theta})\circ\Big(\nabla f_i(\hat{\boldsymbol{\theta}})\otimes\nabla f_j(\hat{\boldsymbol{\theta}})\Big) \\ &+ \frac{1}{2}\mathbb{V}(\boldsymbol{\theta})\circ\Big(f_i(\hat{\boldsymbol{\theta}})Hf_j(\hat{\boldsymbol{\theta}})+f_j(\hat{\boldsymbol{\theta}})Hf_i(\hat{\boldsymbol{\theta}})\Big) \\ &+ \frac{1}{2}\mathbb{S}(\boldsymbol{\theta})\circ\Big(Hf_i(\hat{\boldsymbol{\theta}})\otimes\nabla f_j(\hat{\boldsymbol{\theta}})+Hf_j(\hat{\boldsymbol{\theta}})\otimes\nabla f_i(\hat{\boldsymbol{\theta}})\Big) \\ &+ \frac{1}{4}\mathbb{K}(\boldsymbol{\theta})\circ\Big(Hf_i(\hat{\boldsymbol{\theta}})\otimes Hf_j(\hat{\boldsymbol{\theta}})\Big).\end{aligned} \tag{S6}$$

S1.3.3 Third-order

Next, we take the cube of eq. (S3) to obtain

$$
\begin{aligned}
f^3(\boldsymbol{\theta}) = f^3(\hat{\boldsymbol{\theta}}) &+ 3f^2(\hat{\boldsymbol{\theta}})\nabla f(\hat{\boldsymbol{\theta}})\cdot(\boldsymbol{\theta}-\hat{\boldsymbol{\theta}}) + 3f(\hat{\boldsymbol{\theta}})\left(\nabla f(\hat{\boldsymbol{\theta}})\cdot(\boldsymbol{\theta}-\hat{\boldsymbol{\theta}})\right)^2 \\
&+ \underbrace{\left(\nabla f(\hat{\boldsymbol{\theta}})\cdot(\boldsymbol{\theta}-\hat{\boldsymbol{\theta}})\right)^3}_{\text{Term 1}} + \frac{3}{2}f^2(\hat{\boldsymbol{\theta}})Hf(\hat{\boldsymbol{\theta}})\circ M_2(\boldsymbol{\theta}-\hat{\boldsymbol{\theta}}) \\
&+ 3f(\hat{\boldsymbol{\theta}})\left(\nabla f(\hat{\boldsymbol{\theta}})\cdot(\boldsymbol{\theta}-\hat{\boldsymbol{\theta}})\right)\left(Hf(\hat{\boldsymbol{\theta}})\circ M_2(\boldsymbol{\theta}-\hat{\boldsymbol{\theta}})\right) \\
&+ \frac{3}{2}\underbrace{\left(\nabla f(\hat{\boldsymbol{\theta}})\cdot(\boldsymbol{\theta}-\hat{\boldsymbol{\theta}})\right)^2\left(Hf(\hat{\boldsymbol{\theta}})\circ M_2(\boldsymbol{\theta}-\hat{\boldsymbol{\theta}})\right)}_{\text{Term 2}} \\
&+ \frac{3}{4}f(\hat{\boldsymbol{\theta}})\left(Hf(\hat{\boldsymbol{\theta}})\circ M_2(\boldsymbol{\theta}-\hat{\boldsymbol{\theta}})\right)^2 \\
&+ \frac{3}{4}\underbrace{\left(\nabla f(\hat{\boldsymbol{\theta}})\cdot(\boldsymbol{\theta}-\hat{\boldsymbol{\theta}})\right)\left(Hf(\hat{\boldsymbol{\theta}})\circ M_2(\boldsymbol{\theta}-\hat{\boldsymbol{\theta}})\right)^2}_{\text{Term 3}} \\
&+ \frac{1}{6}\underbrace{\left(Hf(\hat{\boldsymbol{\theta}})\circ M_2(\boldsymbol{\theta}-\hat{\boldsymbol{\theta}})\right)^3}_{\text{Term 4}}.
\end{aligned}
\tag{S7}
$$

As with the second-order expression, we now apply results from propositions 1 to 3 so that expectations that relate to the moments of $\boldsymbol{\theta}$ can be taken.

Term 1

$$
\begin{aligned}
\left(\nabla f(\hat{\boldsymbol{\theta}})\cdot(\boldsymbol{\theta}-\hat{\boldsymbol{\theta}})\right)^3 &= \left(\nabla f(\hat{\boldsymbol{\theta}})\cdot(\boldsymbol{\theta}-\hat{\boldsymbol{\theta}})\right)^2\left(\nabla f(\hat{\boldsymbol{\theta}})\cdot(\boldsymbol{\theta}-\hat{\boldsymbol{\theta}})\right), \\
&= \left(M_2(\boldsymbol{\theta}-\hat{\boldsymbol{\theta}})\circ\left(\nabla f(\hat{\boldsymbol{\theta}})\otimes\nabla f(\hat{\boldsymbol{\theta}})\right)\right)\left((\boldsymbol{\theta}-\hat{\boldsymbol{\theta}})\circ\nabla f(\hat{\boldsymbol{\theta}})\right), \\
&= \left(M_2(\boldsymbol{\theta}-\hat{\boldsymbol{\theta}})\circ M_2\left(\nabla f(\hat{\boldsymbol{\theta}})\right)\right)\left((\boldsymbol{\theta}-\hat{\boldsymbol{\theta}})\circ\nabla f(\hat{\boldsymbol{\theta}})\right), \\
&= \left(M_2(\boldsymbol{\theta}-\hat{\boldsymbol{\theta}})\otimes(\boldsymbol{\theta}-\hat{\boldsymbol{\theta}})\right)\circ\left(M_2\left(\nabla f(\hat{\boldsymbol{\theta}})\right)\otimes\nabla f(\hat{\boldsymbol{\theta}})\right), \\
&= M_3(\boldsymbol{\theta}-\hat{\boldsymbol{\theta}})\circ M_3\left(\nabla f(\hat{\boldsymbol{\theta}})\right).
\end{aligned}
$$

Term 2

$$
\begin{aligned}
\left(\nabla f(\hat{\boldsymbol{\theta}})\cdot(\boldsymbol{\theta}-\hat{\boldsymbol{\theta}})\right)^2\left(Hf(\hat{\boldsymbol{\theta}})\circ M_2(\boldsymbol{\theta}-\hat{\boldsymbol{\theta}})\right) &= \left(M_2\left(\nabla f(\hat{\boldsymbol{\theta}})\right)\circ M_2(\boldsymbol{\theta}-\hat{\boldsymbol{\theta}})\right)\left(Hf(\hat{\boldsymbol{\theta}})\circ M_2(\boldsymbol{\theta}-\hat{\boldsymbol{\theta}})\right), \\
&= \left(M_2\left(\nabla f(\hat{\boldsymbol{\theta}})\right)\otimes Hf(\hat{\boldsymbol{\theta}})\right)\circ\left(M_2(\boldsymbol{\theta}-\hat{\boldsymbol{\theta}})\otimes M_2(\boldsymbol{\theta}-\hat{\boldsymbol{\theta}})\right), \\
&= \left(M_2\left(\nabla f(\hat{\boldsymbol{\theta}})\right)\otimes Hf(\hat{\boldsymbol{\theta}})\right)\circ M_4(\boldsymbol{\theta}-\hat{\boldsymbol{\theta}}).
\end{aligned}
$$

Term 3

$$
\begin{aligned}
\left(\nabla f(\hat{\boldsymbol{\theta}})\cdot(\boldsymbol{\theta}-\hat{\boldsymbol{\theta}})\right)\left(Hf(\hat{\boldsymbol{\theta}})\circ M_2(\boldsymbol{\theta}-\hat{\boldsymbol{\theta}})\right)^2 &= \left(Hf(\hat{\boldsymbol{\theta}})\circ M_2(\boldsymbol{\theta}-\hat{\boldsymbol{\theta}})\right)^2\left(\nabla f(\hat{\boldsymbol{\theta}})\cdot(\boldsymbol{\theta}-\hat{\boldsymbol{\theta}})\right), \\
&= \left(\left(Hf(\hat{\boldsymbol{\theta}})\otimes Hf(\hat{\boldsymbol{\theta}})\right)\circ M_4(\boldsymbol{\theta}-\hat{\boldsymbol{\theta}})\right)\left(\nabla f(\hat{\boldsymbol{\theta}})\cdot(\boldsymbol{\theta}-\hat{\boldsymbol{\theta}})\right), \\
&= \left(\left(Hf(\hat{\boldsymbol{\theta}})\otimes Hf(\hat{\boldsymbol{\theta}})\otimes\nabla f(\hat{\boldsymbol{\theta}})\right)\circ M_5(\boldsymbol{\theta}-\hat{\boldsymbol{\theta}})\right).
\end{aligned}
$$

Term 4

$$
\begin{aligned}
\left(Hf(\hat{\boldsymbol{\theta}}) \circ M_2(\boldsymbol{\theta}-\hat{\boldsymbol{\theta}})\right)^3 &= \left(Hf(\hat{\boldsymbol{\theta}}) \circ M_2(\boldsymbol{\theta}-\hat{\boldsymbol{\theta}})\right)^2 \left(Hf(\hat{\boldsymbol{\theta}}) \circ M_2(\boldsymbol{\theta}-\hat{\boldsymbol{\theta}})\right), \\
&= \left(\left(Hf(\hat{\boldsymbol{\theta}}) \otimes Hf(\hat{\boldsymbol{\theta}})\right) \circ M_4(\boldsymbol{\theta}-\hat{\boldsymbol{\theta}})\right) \left(Hf(\hat{\boldsymbol{\theta}}) \circ M_2(\boldsymbol{\theta}-\hat{\boldsymbol{\theta}})\right), \\
&= \left(Hf(\hat{\boldsymbol{\theta}}) \otimes Hf(\hat{\boldsymbol{\theta}}) \otimes Hf(\hat{\boldsymbol{\theta}})\right) \circ M_6(\boldsymbol{\theta}-\hat{\boldsymbol{\theta}}).
\end{aligned}
$$

At third order, we make the approximation that $\langle M_5(\boldsymbol{\theta}-\hat{\boldsymbol{\theta}})\rangle = \langle M_6(\boldsymbol{\theta}-\hat{\boldsymbol{\theta}})\rangle = \mathbf{0}$; therefore, the expectation of terms 3 and 4 above are also zero. Therefore, we arrive at the following expression for the expectation of the third-order moment

$$
\begin{aligned}
\langle f^3(\boldsymbol{\theta})\rangle = f^3(\hat{\boldsymbol{\theta}}) &+ 3f(\hat{\boldsymbol{\theta}})\mathbb{V}(\boldsymbol{\theta}) \circ M_2\left(\nabla f(\hat{\boldsymbol{\theta}})\right) + \mathbb{S}(\boldsymbol{\theta}) \circ M_3\left(\nabla f(\hat{\boldsymbol{\theta}})\right) \\
&+ \frac{3}{2} f^2(\hat{\boldsymbol{\theta}})\mathbb{V}(\boldsymbol{\theta}) \circ Hf(\hat{\boldsymbol{\theta}}) \\
&+ 3f(\hat{\boldsymbol{\theta}})\mathbb{S}(\boldsymbol{\theta}) \circ \left(Hf_j(\hat{\boldsymbol{\theta}}) \otimes \nabla f_i(\hat{\boldsymbol{\theta}})\right) \\
&+ \frac{3}{2}\mathbb{K}(\boldsymbol{\theta}) \circ \left(M_2\left(\nabla f(\hat{\boldsymbol{\theta}})\right) \otimes Hf(\hat{\boldsymbol{\theta}})\right) \\
&+ \frac{3}{4} f(\hat{\boldsymbol{\theta}})\mathbb{K}(\boldsymbol{\theta}) \circ \left(Hf(\hat{\boldsymbol{\theta}}) \otimes Hf(\hat{\boldsymbol{\theta}})\right).
\end{aligned} \tag{S8}
$$

S1.3.4 Reshaped expressions

We note that eqs. (S4), (S6) and (S8) include only scalar multiplication, the Frobenius inner-product, and the Kronecker product (but no matrix products), all of which are operations that are independent of the matrix shape (for example, we could apply the vec(·) operator to all matrices in eqs. (S4), (S6) and (S8) and the equations would remain valid). Therefore, we introduce a generalisation of the Kronecker product such that moment expressions $M_p(\boldsymbol{\varphi})$ are p-dimensional tensors with elements

$$
\left[M_p(\boldsymbol{\varphi})\right]_{a_1 a_2,\dots,a_p} = \prod_{i=1}^{p} \varphi_{a_i}. \tag{S9}
$$

This formulation allows for the expectation $\langle M_p(\boldsymbol{\theta}-\hat{\boldsymbol{\theta}})\rangle$ to be formulated more easily element-by-element for common distributions, such as when $\boldsymbol{\theta}$ is multivariate normal, or Gamma distributed.

Definition 4. (Multidimensional Kronecker product) *For matrices $A \in \mathbb{R}^{m\times n}$ and $B \in \mathbb{R}^{p\times q}$, the multidimensional Kronecker product $\overline{\otimes}$ of A and B*

$$
C = A \,\overline{\otimes}\, B \tag{S10}
$$

such that

$$
C_{1:m,1:n,i,j} = B_{i,j}A. \tag{S11}
$$

The multidimensional Kronecker product is similarly applicable to A and B as m- and p-dimensional tensors.

Definition 5. (Kronecker power) *For tensor A and positive integer n, the Kronecker power is*

$$A^{\overline{\otimes}\, n} = \underbrace{A \mathbin{\overline{\otimes}} A \mathbin{\overline{\otimes}} \cdots \mathbin{\overline{\otimes}} A}_{n \text{ times}}. \tag{S12}$$

From these definitions, we can redefine

$$M_p(\boldsymbol{\varphi}) = \boldsymbol{\varphi}^{\overline{\otimes}\, p}. \tag{S13}$$

and writing eqs. (S4), (S6) and (S8) using definitions 4 and 5 allows us to arrive at the following expressions.

$$\langle f_i(\theta) \rangle \approx f_i(\hat{\boldsymbol{\theta}}) + \mathbb{V}(\boldsymbol{\theta}) \circ \frac{1}{2} H f_i(\hat{\boldsymbol{\theta}}), \tag{S14}$$

$$\begin{aligned} \langle f_i^2(\theta) \rangle \approx f_i^2(\hat{\boldsymbol{\theta}}) &+ \mathbb{V}(\boldsymbol{\theta}) \circ \Big(\nabla f_i(\hat{\boldsymbol{\theta}})^{\overline{\otimes}\, 2} + f_i(\hat{\boldsymbol{\theta}}) H f_i(\hat{\boldsymbol{\theta}}) \Big) \\ &+ \mathbb{S}(\boldsymbol{\theta}) \circ \Big(H f_i(\hat{\boldsymbol{\theta}}) \mathbin{\overline{\otimes}} \nabla f_i(\hat{\boldsymbol{\theta}}) \Big) \\ &+ \mathbb{K}(\boldsymbol{\theta}) \circ \frac{1}{4} H f_i(\hat{\boldsymbol{\theta}})^{\overline{\otimes}\, 2}, \end{aligned} \tag{S15}$$

$$\begin{aligned} \langle f_i^3(\theta) \rangle \approx f_i^3(\hat{\boldsymbol{\theta}}) &+ \mathbb{V}(\boldsymbol{\theta}) \circ \frac{3}{2} f_i(\hat{\boldsymbol{\theta}}) \Big(2 \nabla f_i(\hat{\boldsymbol{\theta}})^{\overline{\otimes}\, 2} + f_i(\hat{\boldsymbol{\theta}}) H f_i(\hat{\boldsymbol{\theta}}) \Big) \\ &+ \mathbb{S}(\boldsymbol{\theta}) \circ \Big(\nabla f_i(\hat{\boldsymbol{\theta}})^{\overline{\otimes}\, 3} + 3 f_i(\hat{\boldsymbol{\theta}}) H f_i(\hat{\boldsymbol{\theta}}) \mathbin{\overline{\otimes}} \nabla f_i(\hat{\boldsymbol{\theta}}) \Big) \\ &+ \mathbb{K}(\boldsymbol{\theta}) \circ 3 \Big(\frac{1}{4} f_i(\hat{\boldsymbol{\theta}}) H f_i(\hat{\boldsymbol{\theta}})^{\overline{\otimes}\, 2} + \frac{1}{2} \nabla f_i(\hat{\boldsymbol{\theta}})^{\overline{\otimes}\, 2} \mathbin{\overline{\otimes}} H f_i(\hat{\boldsymbol{\theta}}) \Big). \end{aligned} \tag{S16}$$

and

$$\begin{aligned} \langle f_i(\boldsymbol{\theta}) f_j(\boldsymbol{\theta}) \rangle \approx\ & f_i(\hat{\boldsymbol{\theta}}) f_j(\hat{\boldsymbol{\theta}}) \\ &+ \mathbb{V}(\boldsymbol{\theta}) \circ \frac{1}{2} \Big(f_i(\hat{\boldsymbol{\theta}}) H f_j(\hat{\boldsymbol{\theta}}) + f_j(\hat{\boldsymbol{\theta}}) H f_i(\hat{\boldsymbol{\theta}}) + 2 \nabla f_i(\hat{\boldsymbol{\theta}}) \mathbin{\overline{\otimes}} f_j(\hat{\boldsymbol{\theta}}) \Big) \\ &+ \mathbb{S}(\boldsymbol{\theta}) \circ \Big(\nabla f_i(\hat{\boldsymbol{\theta}}) \mathbin{\overline{\otimes}} H f_j(\hat{\boldsymbol{\theta}}) + \nabla f_j(\hat{\boldsymbol{\theta}}) \mathbin{\overline{\otimes}} H f_i(\hat{\boldsymbol{\theta}}) \Big) \\ &+ \mathbb{K}(\boldsymbol{\theta}) \circ \frac{1}{4} H f_i(\hat{\boldsymbol{\theta}}) \mathbin{\overline{\otimes}} H f_j(\hat{\boldsymbol{\theta}}). \end{aligned} \tag{S17}$$

S2 Kolmogorov-Smirnov test results for Fig 2

In Fig 2 of the main document we compare approximate solutions to the random parameter logistic model to a kernel density estimate constructed from $N = 10^5$ samples. Here, we compute p-values from a Kolmogorov-Smirnov test that compares N_i samples to each approximate distribution (the null hypothesis is that samples are drawn from the approximate distribution). For this model, both approximations perform well, with no evidence at the $\alpha = 0.05$ level to reject the null hypothesis for $N_i \leq 100$, with the gamma approximation providing no evidence to reject the null hypothesis for $N_i \leq 1000$.

Table A

		N_i				
	Distribution	10	100	1000	10 000	100 000
(a)	Normal	0.507	0.173	0.660	1.69×10^{-3}	3.28×10^{-39}
	Gamma	0.436	0.636	0.995	0.103	9.67×10^{-4}
(b)	Normal	0.132	0.343	0.0936	3.89×10^{-10}	1.37×10^{-90}
	Gamma	0.609	0.588	0.270	0.244	0.98
(c)	Normal	0.345	0.163	0.00989	3.15×10^{-39}	5.31×10^{-298}
	Gamma	0.124	0.569	0.196	6.22×10^{-3}	5.87×10^{-21}

S3 Approximate solutions to random parameter logistic model

Here, we consider dependent observations of the logistic model, such that

$$\boldsymbol{f}(\boldsymbol{\theta}) = \begin{bmatrix} f_1(\boldsymbol{\theta}) \\ f_2(\boldsymbol{\theta}) \\ f_3(\boldsymbol{\theta}) \end{bmatrix} = \begin{bmatrix} r(20;\boldsymbol{\theta}) \\ r(30;\boldsymbol{\theta}) \\ r(40;\boldsymbol{\theta}) \end{bmatrix}, \tag{S18}$$

where $r(t;\boldsymbol{\theta})$ is the solution to the logistic model. We assume that $\boldsymbol{\theta} = \left[r_0, \lambda, R\right]^\intercal$ are correlated random parameters with density function

$$\boldsymbol{\theta} \sim \text{MVN}\left(\begin{bmatrix} \mu_\lambda \\ \mu_R \\ \mu_{r_0} \end{bmatrix}, \begin{bmatrix} \sigma_\lambda^2 & \rho\sigma_\lambda\sigma_R & 0 \\ \rho\sigma_\lambda\sigma_R & \sigma_R^2 & 0 \\ 0 & 0 & \sigma_{r_0}^2 \end{bmatrix} \right). \tag{S19}$$

In Fig A we compare approximate solutions based on normal (two-moment) and gamma (three-moment) distributions for $\mu_\lambda = 0.5$, $\mu_R = 300$, $\mu_{r_0} = 10$, $\sigma_\lambda = 0.05$, $\sigma_R = 50$, $\sigma_{r_0} = 1$, $\rho = 0.8$.

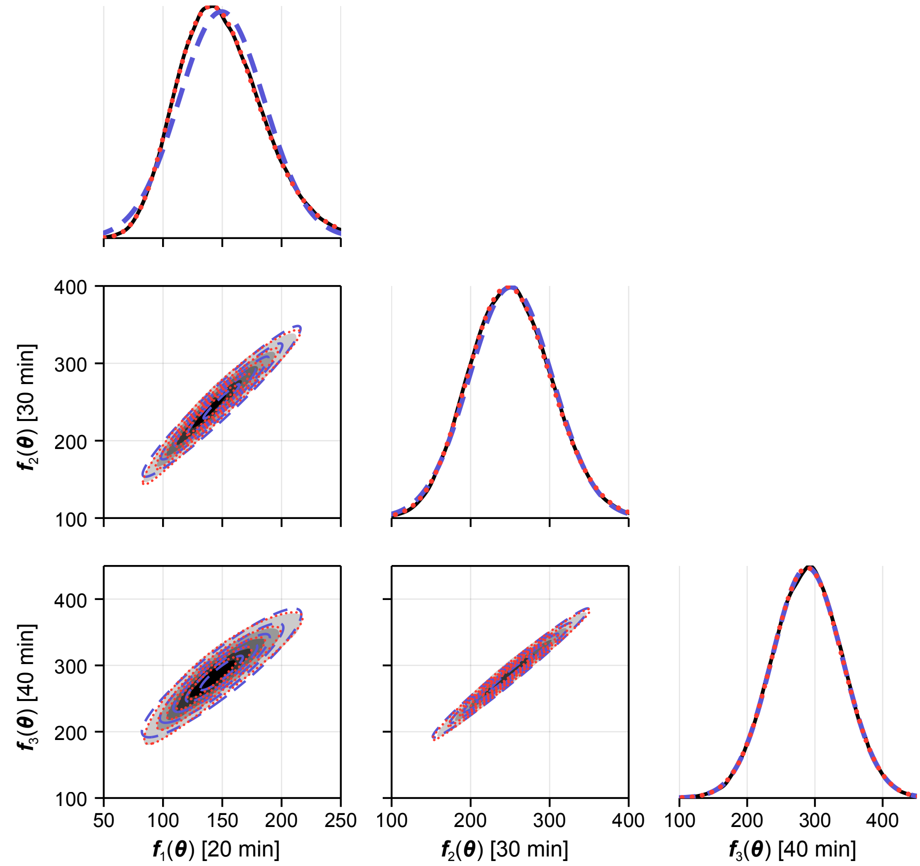

Fig A. Approximate transformation for dependent observations of the logistic model. The model output, $\boldsymbol{f}(\boldsymbol{\theta}) \in \mathbb{R}^3$, comprises observations of the logistic model at $t = 20$, 30 and 40 min. Shown is synthetic data from 10^5 samples of $\boldsymbol{\theta}$ and an approximate transformed distribution based on the multivariate normal distribution (blue dashed) and multivariate Gamma distribution (red dotted).

S4 Approximate solutions for linear two-pool model

In Fig B we compare the gamma approximation of the solution of the random parameter linear two-pool model to a kernel density estimate constructed through simulation. In Table B we compute p-values from a Kolmogorov-Smirnov test that compares N_i samples to the approximate distribution (the null hypothesis is that samples are drawn from the approximate distribution). For this model, the approximation performs well at the at the $\alpha = 0.05$ level in all cases.

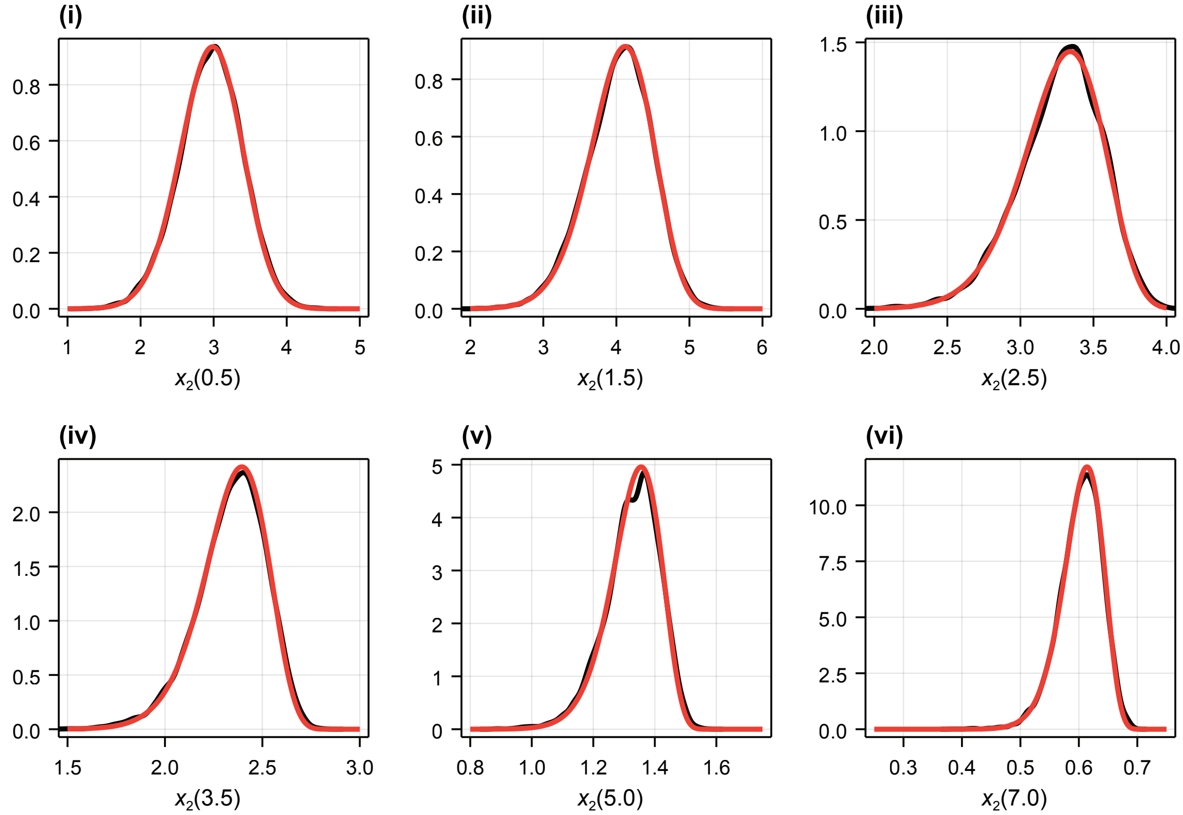

Fig B. Approximate transformation for independent observations of the linear two-pool model. We compare the accuracy of an approximate solution based on a three-moment-matched gamma distribution (red). Also shown are kernel density estimates (black) produced from 10^4 samples.

Table B

		N_i			
	Time	10	100	1000	10 000
(a)	0.5	0.889	0.947	0.315	0.278
(b)	1.5	0.987	0.660	0.508	0.720
(c)	2.5	0.223	0.0754	0.814	0.0787
(d)	3.5	0.205	0.284	0.101	0.262
(e)	5.0	0.195	0.700	0.113	0.662
(f)	7.0	0.753	0.857	0.296	0.407

S5 Approximate solutions for non-linear two-pool model

In Fig C we compare the bivariate gamma approximation of the solution of the random parameter non-linear two-pool model to a kernel density estimate constructed through simulation. In Table C we compute p-values from a Kolmogorov-Smirnov test that compares N_i samples to the approximate marginal distribution (the null hypothesis is that samples are drawn from the approximate marginal distribution). For this model, the approximation performs well at the at the $\alpha = 0.05$ level in most cases for $N_i \leq 1000$.

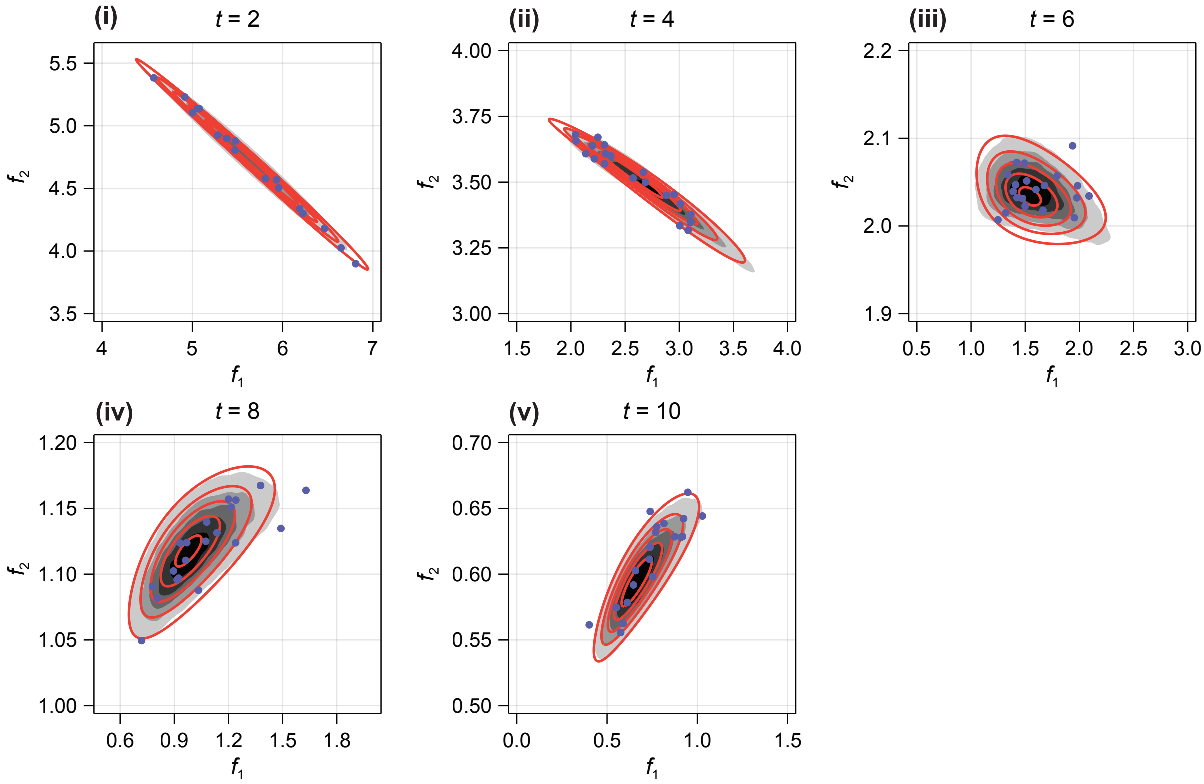

Fig C. Approximate transformation for dependent observations of the non-linear two-pool model. We compare the accuracy of an approximate solution based on a three-moment-matched gamma distribution (red). Also shown are kernel density estimates (greyscale) produced from 10^5 samples and synthetic data used for analysis (blue).

Table C

		N_i							
		10		100		1000		10 000	
	Time	f_1	f_2	f_1	f_2	f_1	f_2	f_1	f_2
(a)	2.0	0.506	0.240	0.421	0.762	0.699	0.0806	0.140	0.196
(b)	4.0	0.708	0.0273	0.515	0.917	0.572	0.362	0.205	0.326
(c)	6.0	0.285	0.169	0.423	0.380	0.825	0.0525	0.499	8.09×10^{-7}
(d)	8.0	0.162	0.579	0.220	0.233	0.167	0.235	0.0186	0.0043
(e)	10.0	0.420	0.438	0.542	0.748	0.594	0.401	0.210	0.921

S6 Additional comparison for misspecified bimodal model

Here, we provide additional results exploring how misspecification of a parameter distribution affects identifiability and model predictions. We consider a case where λ has a bimodal distribution, given by a normal mixture $\lambda \sim w\lambda_1 + (1-w)\lambda_2$ where

$$\begin{aligned} \lambda_1 &\sim \mathcal{N}\left(\mu_\lambda^{(1)}, \sigma_\lambda^{(1)}\right), \\ \lambda_2 &\sim \mathcal{N}\left(\mu_\lambda^{(2)}, \sigma_\lambda^{(2)}\right). \end{aligned} \tag{S20}$$

A similar problem was previously explored by Banks et al. [3]. In the main text, we set $\mu_\lambda^{(1)} = 0.9$, $\mu_\lambda^{(1)} = 1.1$, $\sigma_\lambda^{(1)} = \sigma_\lambda^{(2)} = 0.05$ and $w = 0.4$. Here, we explore a case where the subpopulations are more distinct, setting $\mu_\lambda^{(1)} = 0.7$, $\mu_\lambda^{(1)} = 1.3$ (Fig D(i)). Results are shown in Fig D.

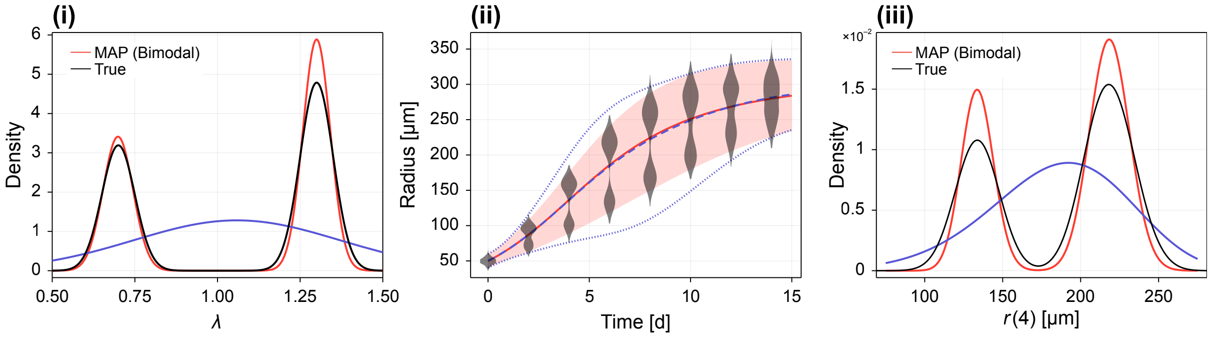

Fig D. Inference and prediction where parameter distribution is misspecified. We explore a case where the underlying growth rate distribution has a bimodal distribution, modelled as the mixture $w\lambda_1 + (1-w)\lambda_2$ with $\lambda_1 = \mathcal{N}(0.9, 0.05^2)$, $\lambda_2 = \mathcal{N}(1.1, 0.05^2)$ and $w = 0.5$. To ensure practical identifiability, we use a large sample size of $n = 1000$ per time point. In (i) we compare the true distribution (black) to a MAP prediction (equivalent to MLE) based on the true bimodal distribution (orange) and a misspecified normal distribution (blue). In (ii), predictions at the MAP estimates are compared to the data. A 95% prediction interval is shown for the true model (shaded) and the misspecified model (blue dashed), solid curves to the mean, and violin plots show the data. In (iii), we compare predictions for the density from the true and misspecified models at $t = 4$.

S7 Bistable model

Here, we consider an extension of the logistic model, known as the strong Allee effect,

$$\frac{\mathrm{d}r}{\mathrm{d}t} = \frac{\lambda}{3} r \left(\frac{r}{A} - 1\right)\left(1 - \frac{r}{R}\right), \qquad r(0) = r_0. \tag{S21}$$

Whereas the standard logistic model has a single unstable steady-state at $r = 0$ and a single stable steady-state at $r = R$, the logistic model with strong Allee effect has two stable steady-states at $r = 0$ and $r = R$, and an unstable steady state at $r = A$. In effect, solutions to eq. (S21) with $r_0 < A$ become extinct, $r \to 0$ and solutions with $r_0 > A$ grow to carrying capacity $r \to R$ (Fig D(i)).

To demonstrate a case where our approximate should not be used, consider model eq. (S21) with a single random parameter,

$$r_0 \sim \mathcal{N}(51, 1), \tag{S22}$$

and with constant parameters $\lambda = 3$, $R = 300$, $A = 50$. Therefore, we expect approximately 84% of realisations to grow to carrying capacity, and 16% to tend to extinction (Fig E(i)). For t sufficiently large, the distribution of $r(t)$ is bimodal and constrained between $0 < r(t) < R$. However, this behaviour cannot be captured by our approximate solution, which uses information about the derivatives of $r(t)$ only at $r_0 = 51$ (Fig E).

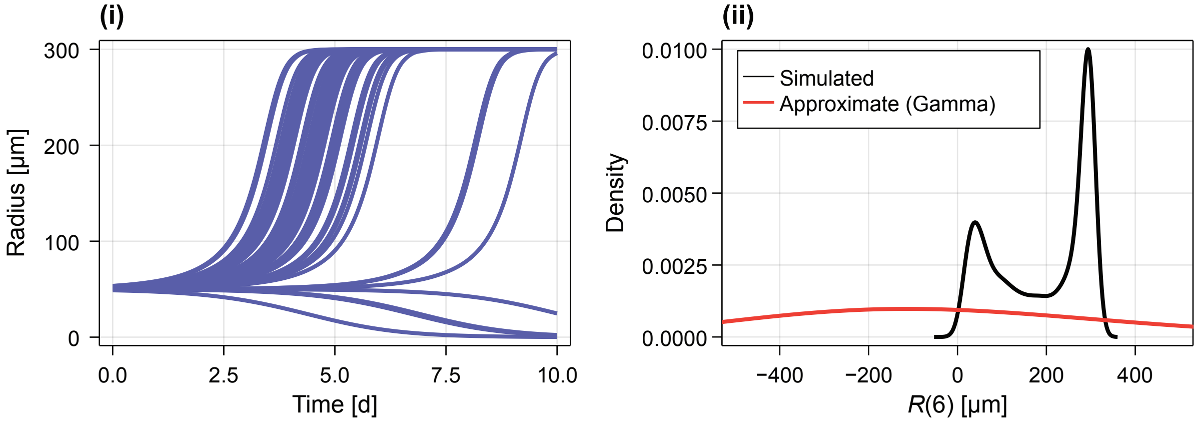

Fig E. Failure of approximate solution for a bistable model. (i) Solutions to eq. (S21) for 50 random parameter combinations. (ii) Distribution of $r(5)$, showing results from 10^4 simulated trajectories (black) and an approximate solution based on a gamma distribution (red).

S8 Uniformly distributed parameters model

Here, investigate the ability of our method to infer parameter distributions that are not well described by their moments by reproducing analysis in Section 3.1.1 for the logistic model where the parameter distributions are uniform. We set

$$\begin{aligned} r_0 &\sim \text{Uniform}\left(\mu_{r_0} - \sqrt{3}\sigma_{r_0}, \mu_{r_0} + \sqrt{3}\sigma_{r_0}\right), \\ \lambda &\sim \text{Uniform}\left(\mu_\lambda - \sqrt{3}\sigma_\lambda, \mu_\lambda + \sqrt{3}\sigma_\lambda\right), \\ R &\sim \text{Uniform}\left(\mu_R - \sqrt{3}\sigma_R, \mu_R + \sqrt{3}\sigma_R\right), \end{aligned} \tag{S23}$$

where we parameterise each distribution in terms of a mean and variance parameter. To ensure model parameters are identifiable, we neglect measurement noise in this example. Hyperparameters are otherwise set to match those in the main paper $\mu_\lambda = 1$, $\mu_R = 300$, $\mu_{r_0} = 50$, $\sigma_\lambda = 0.05$, $\sigma_R = 20$ and $\sigma_{r_0} = 3$.

In Fig F(i) we compare the approximate solutions to the random parameter logistic model at $t = 2\,\text{d}$ to a kernel density estimate produced from 10^5 samples. The approximations are poor in comparison to those in the main paper (Fig 2) for the case where model parameters are normally distributed. However, both approximations recapture the mean and variance of the simulated data.

Next, we perform profile likelihood analysis to establish the identifiability of μ_R and σ_R from $N = 10$ measurements at each $t = 0, 2, 4, ..., 14$. Results in Fig F(ii–iii) demonstrate that both parameters are identifiable, and that despite the discrepancy between the approximate and simulated distributions in Fig F(i) we are able to recover the true value of each parameter.

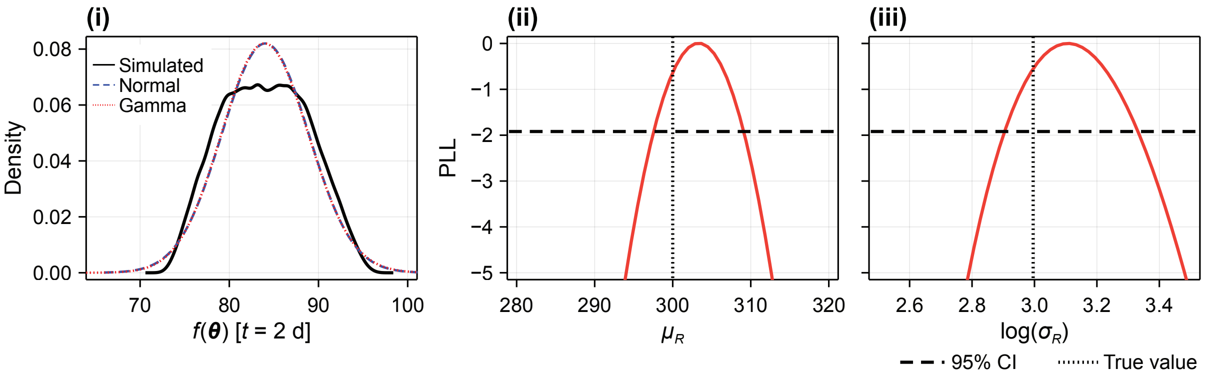

Fig F. Analysis of logistic model with uniformly distributed random parameters. (i) Comparison of the normal (blue dashed) and gamma (red dotted) approximations to a kernel density estimate produced using 10^5 samples. (ii–iii) Profile likelihood results for μ_R and $\log(\sigma_R)$ from $N = 10$ samples from each $t = 0, 2, 4, ..., 14$ using the gamma approximation. Shown are likelihood profiles (red), the true value used to produce synthetic data (vertical dotted), and the threshold for an approximate 95% confidence interval.

References

[1] Lee, G Seber and A , Lee A. Linear Regression Analysis. John Wiley and Sons; 2002.

[2] Petersen KB, Pedersen MS. The Matrix Cookbook. Technical University of Denmark. 2012;.

[3] Banks HT, Meade AE, Schacht C, Catenacci J, Thompson WC, Abate-Daga D, et al. Parameter estimation using aggregate data. Applied Mathematics Letters. 2020;100:105999. doi:10.1016/j.aml.2019.105999.
