# Supplementary material for: Efficient inference and identifiability analysis for differential equation models with random parameters
Source: PLoS Comput Biol. 2022 Nov 28;18(11):e1010734. doi: 10.1371/journal.pcbi.1010734 (PMC9731444; doi:10.1371/journal.pcbi.1010734)
Supplement: Attachment — Submitted filename: letter.pdf [file pcbi.1010734.s002.pdf]

Date October 26, 2022
Contact Matthew J. Simpson
Phone +61 4 1369 6607
E-mail matthew.simpson@qut.edu.au
Subject Manuscript PCOMPBIOL-D-22-01128

Matthew J. Simpson

School of Mathematical Sciences
Science and Engineering Faculty
Queensland University of Technology
GPO Box 2434, GP Campus
Brisbane, Queensland 4001 Australia

Professor Ricardo Martinez-Garcia
Academic Editor
PLOS Computational Biology

Dear Professor Martinez-Garcia,

I am writing to submit a revised manuscript, "Efficient inference and identifiability analysis for differential equation models with random parameters" that has been accepted for publication as a methods article in *PLOS Computational Biology* pending minor revisions

We thank all three referees for their positive and helpful comments, and are pleased to see that all three recommend the paper for publication. In response to the comments from referee 1, we have clarified technical details relating to our approach, and provided additional results to demonstrate that our methodology can still be robust to the forms of misspecification that we demonstrate. Noting that both referees 1 and 3 commented on the dimensionality of the state-space in our three didactic examples, we have added text to the manuscript to discuss the key assumption behind our method: namely, that data of any dimension are approximately jointly normally distributed, or that data of dimension less than two can be described approximately using shifted gamma distributions that account for skew.

To address the concerns of referee 2, in the revised manuscript we provide an expanded and explicit comparison of our contribution to that of others, particularly the research work suggested by the referee. We also now explicitly provide an expression for the log-likelihood function, to make the methodology clear.

We attach to this letter a point-by-point response to the specific comments raised by each referee. Changes to the manuscript are indicated in the margins and highlighted in blue font. We hope that you find this revised manuscript suitable for publication in *PLOS Computational Biology*.

Thank you for your consideration.

Yours sincerely,

Matthew J. Simpson

Referee 1

Browning et al. present a new approach that enables parameter estimation and identifiability analysis for differential equation models incorporating heterogeneity. This approach allows for efficient inference and identifiability analysis by leveraging an approximate solution to the differential equation. Overall this work is widely applicable to differential equation models that explicitly model heterogeneity observed in the data via random parameters with parametric distributions. The efficient approach presented in this work enables critical analyses that would otherwise be computationally intractable for many applications.

Overall, I found that the authors clearly explain their method and provide adequate background and detail to enable a reader's understanding. Additionally, the authors use three test cases to provide an excellent introduction to the identifiability analysis of this class of models. I believe this work will allow future users to conduct a similar analysis of their models.

I have no major changes to suggest, but I have a few minor points I would like the authors to address:

Response: We thank the referee for their positive review of our manuscript and now addressed their minor comments.

R1.1 The proposed approach requires the user to specify parametric distributions for the model parameters. They show that misspecification of these distributions can lead to errors in the identifiability analysis. I am concerned about the limitations of this approach when prior knowledge of these distributions is limited. Can the authors address the possible limitations of assuming specific parametric distributions for model parameters and provide suggestions for when the distribution may be unknown?

Response: We agree that misspecification of the mathematical model (or specifically, the distributional form of the model parameters) can give rise to misleading results. However, although the way we present the methodology involves assuming a specific distributional form for the model parameters, it is the moments of the input parameters are inferred that are inferred. We now explicitly note this in the revised manuscript (Page 3). Further, we note that when we explore misspecification by fitting a univariate normal distribution where the true distribution is a bivariate mixture (fig. 5, Page 13), the mean and variance are both recovered accurately (1.016 vs 1.020 for the inferred and true mean, respectively; 1.21×10^{-2} vs 1.22×10^{-2} for the inferred and true variance, respectively) (Page 13).

R1.2 How does the identifiability of a model parameter affect the identifiability of its distributions hyper-parameters? For instance, if one found that a parameter is non-identifiable via an a priori analysis, what should they expect to see in the identifiability analysis of that parameter's hyper-parameters?

Response: If a model parameter was a priori established as non-identifiable (using structural identifiability analysis, for example) we *hypothesise* that the *mean* of the model parameter distribution would likely also be non-identifiable. It is more difficult to extrapolate what one might expect for the variance of the model parameter distribution: in many cases, we expect that this might also be non-identifiable in general, but that we may still be able to establish an upper bound on the variance based on the variance of the output.

Given that our expression for the output average (eq. (11), Page 6) includes terms relating to both the parameter mean and variance, it is difficult to comment on the referee's question with enough certainty to be stated in the manuscript. However, the expressions linking the input and output moments that we provide might allow more rigorous analysis of such questions in the future. We now state this in the revised manuscript (Page 17).

R1.3 Does the identifiability of a parameter's variance terms provide insight into the model's sensitivity to those parameters? For instance, in section 3.1.1, the authors state that the slight identifiability of the variance parameter for lambda indicates that variability in this lambda cannot be distinguished from measurement noise. Does this imply that the model is not particularly sensitive to lambda's value?

Response: Not necessarily. Our approximations for the output variance are similar to those used for traditional sensitivity analysis. Such sensitivity analysis might investigate a first-order map from the variance of a model parameter to the variance of the output. However, this analysis is naturally a forwards problem. The identifiability of model variance is an inverse problem, and while we demonstrate that in some cases one can establish an upper bound on the variance of a parameter, this finding does not relate just to the sensitivity of the model to the parameter value. Given that we find $\mathbb{E}(\lambda)$ to be identifiable in the examples we present, we do expect the model to be sensitive to the value of λ.

R1.4 Can the authors clarify how they determine if a parameter is identifiable from its 95% credible interval in the cases where they perform a Bayesian analysis? For example, in section 3.1.3, lines 393-395, the authors conclude that the distribution of parameter lambda is identifiable. However, it is unclear how they arrived at this conclusion from the results presented in figure 5.

Response: In fig. 5 (Page 13) we establish identifiability of the distribution of λ by examining a 95% credible interval for the density function of λ. In this case, we find that the density function is identified to a tightly constrained region, and so conclude that the distribution of λ is identifiable. We have adjusted the text to make this clear (Page 13).

R1.5 The authors present their method with examples that have relatively few state variables and parameters. Can the authors comment on how this approach scales to models with greater numbers of parameters?

Response: While we work with examples with a relatively small number of state variables and parameters, we note that this is fairly common in mathematical biology (e.g. Hasenauer et al. 2014 *PLOS Comp Biol*). Aside from an increased computational cost as the number of states increases (due to the number of elements in the Hessian matrices, for instance) and the number of parameters increases (due to the number of elements in parameter moment tensors), there is nothing preventing application of our method to compute output moments for any number of state variables. The primary limitation of our method arises in constructing a distributional approximation to the likelihood, since only a multivariate normal approximation can be constructed for cases with more than two state variables. Therefore, the question of state-space dimensionality is a question of whether the observed output distributions are well approximated by a multivariate

normal distribution; or, indeed, transformations of the output distributions. We now comment on these limitations and the potential for our method to handle transformations of the output distributions in the revised paper (Page 3).

R1.6 In addition to these points, I found several typos while reading the manuscript. Can the authors please correct the following typos and double-check the manuscript and supplemental materials:

(a) The caption for figure 4 states "ω_λ = -1.5" however, the corresponding text on line 357 states "ω_λ = 1.5." I believe the value on line 357 needs to be negative.

(b) Line 437 says, "second tool." I believe this should read "second pool."

Response: We have addressed these typos and thoroughly proofread the manuscript.

Referee 2

In their manuscript titled "Efficient inference and identifiability analysis for differential equation models with random parameters", Browning and colleagues introduce a new method for the calibtration of ODE models with random parameters. The model can be used for the description and inference of inter-individual heterogeneity, which is a very relevant problem in the current literature. The proposed method is novel in so far as that noise model is incorporated into the model-transformed random variable, and that the taylor approximation is applied differently than in similar methods such as the method of moments or van kampens system size expansion, which brings some advantages in terms of scalability (potentially at the cost of some accuracy). The paper is very well written and easy to follow, but there are some technical aspects that remain opaque (which I will go into more detail below). I generally like the approach, and definitely think that this paper should be published. However, I am some concerns that I describe below, but I have no doubt the authors will be able to address them.

Response: We thank the reviewer for their overall positive and helpful review of our manuscript, and we now address their specific comments.

R2.1 Embedding in the existing literature:

The paper is a bit heavy on statistical jargon which might make it difficult for readers with a stronger biological background to follow the paper. Specifically it would be great to give a bit more explanation about what the authors mean by random/fixed effects models. I am familiar with these terms in the context of Non-linear mixed effects models, which are likely to be what the authors call "hierarchical" models. Such models have been used in the context of biological models (see e.g., `https://doi.org/10.1186/s12918-015-0203-x`, `https://doi.org/10.1371/journal.pcbi.1004706`, `https://doi.org/10.1371/journal.pone.0124050`, `https://doi.org/10.1038/s41540-018-0079` and references therein). Similarly the authors should contrast their approach to similar approaches such as `https://doi.org/10.1016/j.cels.2018.12.007` or `https://doi.org/10.1038/nmeth.2794`.

Another range of approaches that seems to be relevant, but isn't really discussed are the moment closure approximations (`https://doi.org/10.1063/1.3454685`) or van Kampens system size expansion (`https://doi.org/10.1063/1.3454685`). Both

use taylor expansion to approximate moments, but with respect to different variables, which would be helpful if mentioned in the paper. Usually these methods are employed for the description of stochastic models, but, using the approach described in `https://doi.org/10.1016/j.cels.2018.04.008` which the authors also use, can also be applied to describe heterogeneity. Accordingly, the authors also should contrast their approach to (`https://doi.org/10.1371/journal.pcbi.1005030`) where moment closure and van Kampens approximation are used for parameter inference (in a stochastic modeling context, not a heterogeneity context, but the transfer is trivial.).

Response: We agree, one of the challenges with the random parameter models we deal with is that terminology is fragmented in different parts of the literature. We have now expanded text in the introduction to explicitly mention non-linear mixed effects models that are arguably, as the referee suggests, more common in the systems biology literature (Page 3). Furthermore, we now provide a more explicit comparison between our method and the moment closure and system size expansion approaches in the revised manuscript (Page 3).

R2.2 Snapshot vs Timecourse data:

The authors just briefly mention the issue of considering snapshot vs timecourse data (it would be good to mention these terms such that a more biological audience can also follow) in l142-145. However, this isn't really picked up in the remainder of the manuscript, but is quite relevant in practical terms. The key difference for timecourse data is that there is temporal correlation between simulations across timepoints. It is unclear to me how this is accounted for in the method that the authors present, as what the authors describe only looks like an rearrangement of indices.

Response: We have expanded our explanation of how time-course data can be incorporated in our method with a more concrete example (Page 4).

R2.3 Likelihood function:

I am still unsure whether I am fully grasping what the authors are actually doing. For me it would be quite helpful to have some visual depiction of how the approximation method that the authors are proposing actually works. Similarly, it would probably to explicitly write down the equation for the likelihood pf(y,xi). My understanding is that this would simply, in the case of the normal approach, be a multivariate normal probability density function with mean and standard deviation according to equations 10 and 11. I understand that the equations would be quite bulky, but (10) - (13) already pose a solid chance of scare the reader away ;).

Response: We have now provided an example expression for the full likelihood function $p_{\mathbf{f}}^{(i)}(\cdot)$ at the end of equation 2.2 (Page 8).

R2.4 Approximation Error:

The authors really only discuss the approximation error in figure 2 and the discussion and seem to forget about in the remainder of the results. It would be good to see some more investigation of the approximation error of the method in the more complex settings, to make sure that the findings are not the result of approximation errors.

Response: We agree that the manuscript would benefit from a more formal comparison between the simulated data and each approximate distribution. We now provide such a comparison using the Kolmogorov-Smirnov test for all models in the supporting material (tables S1, S2 and S3). Further, we note that we always take care in the manuscript to compare the fitted model to the data.

R2.5 Minor Points:

(a) I am not sure what the argument about the inverse of f in line 149 is about. In the vast majority of cases f(theta) wont be available analytically because f itself is not available analytically in the first place.

Response: In the revised manuscript, we clarify that the availability of the inverse is required for the density of $\boldsymbol{f}(\boldsymbol{\theta})$ to be calculated directly (as the referee states, the mention of the existence of analytical solutions is likely not relevant to many use cases) (Page 5).

(b) l234 should probably read "log-likelihood function" instead of "likelihood function"

Response: We have addressed this (Page 8) and thoroughly proofread the revised manuscript.

(c) Panel labels in the legend to figure 5 seem misarranged

Response: We have addressed this (Figure 5 caption, Page 13).

(d) For the parameterization of the covariance matrix $D(\xi)$ the authors may want to consult `https://doi.org/10.1016/j.celrep.2021.109507` for some pointers on how to avoid overparameterization

Response: We thank the referee for bringing the novel parameterisation approach of Adlung et al. to our attention, and cite the work in reference to parameterising the mean and covariances of a multivariate normal distribution (Page 7).

(e) Non-identifiability and sloppiness are not the same, see `https://doi.org/10.1016/j.mbs.2016.10.009`

Response: While we maintain that our approach to both local-identifiability and sloppiness analysis using the Fisher information matrix (FIM) is valid, we have removed discussion around model sloppiness to improve the flow of the manuscript (Page 17).

(f) It's a bit uncommon to introduce new data in the discussion, the authors may want to create a separate section discussing benchmarking and the model with a strong Allee effect

Response: Our intention of the Allee effect example is to provide a tangible, however nonessential, example of why our method is applicable only to data that are approximately normal or gamma distributed. We now make this requirement clear in the main text (Page 3), however maintain that the discussion is an appropriate location for these results.

Referee 3

Browning et al present methods for the analysis of models with intrinsic variability in their parameters. Sources of noise in biology are ubiquitous, complex, and often neglected or at least under-appreciated in systems biology analyses of model identifiability and parameter inference. This is thus an important and timely contribution to the literature that will be widely useful. The inclusion of open-source code in Julia is an additional strength of the manuscript. I have only minor requests/suggestions for possible improvement to the paper. These are given below.

Response: We thank the referee for their favourable review of our manuscript, and address their relatively minor concerns below.

R3.1 While the analysis of how skewness or bimodality affect identifiability is interesting, at least in my experience, a far more common occurrence in biological modeling is the choice of a prior that is particularly uninformative (e.g. uniform over large interval). Would such a prior choice affect the inference/identifiability results, and how? This would be a useful example.

Response: We interpret the referees comment on prior choice to be in reference to the distributional forms (normal, bimodal normal mixture, etc) of the model parameters, which can be referred to as the model parameter priors in hierarchical modelling.

The referee raises an interesting point that was not discussed in the original manuscript. As our approach is based on matching and inferring the moments of the input and output distributions, respectively, it is unclear how well our method can approximate distributions that are not well described by their moments (uniform distributions, for instance). To investigate this, we provide additional results in the supporting material that reproduce fig. 2 and fig. 3 where the parameter distributions are uniform. Despite some mismatch between the simulated and approximate model solution, in this case, we are still able to accurately recover the mean and variance of the unknown uniform distributions (Supplementary Material, Page 17; Manuscript, Page 12).

R3.2 I appreciate the advantages of & the need to strive for model simplicity, however, practically, two species is really a lower bound for realistic model sizes in sys bio. I think it would be really beneficial if an example (or even just some discussion without an example) of a larger (e.g. 3 species) model, in light of the analyses contained in this work: studying identifiability with random parameters & the impact of prior choice, etc.

Response: Please refer to our response to R1.5.

R3.3 In Fig 9, please could you improve the caption/description: it is a figure containing quite a lot of details and it is currently hard to figure out several details. Are colors indep MCMC chains? The different dashed/dotted lines in A are hard to distinguish. I cannot see orange solid lines in B. Also what does grey represent in B?

Response: We have modified the text in the caption to fig. 9 to make the interpretation of the figure clear. Further, we have modified the line styles in fig. 9a and fig. 9b to make the lines easier to distinguish (Page 17).
